# Spectral discrimination of breast pathologies *in situ* using spatial frequency domain imaging

**DOI:** 10.1186/bcr3455

**Published:** 2013-08-05

**Authors:** Ashley M Laughney, Venkataramanan Krishnaswamy, Elizabeth J Rizzo, Mary C Schwab, Richard J Barth, David J Cuccia, Bruce J Tromberg, Keith D Paulsen, Brian W Pogue, Wendy A Wells

**Affiliations:** 1 Thayer School of Engineering, Dartmouth College Hanover, Hanover NH 03755, USA; 2 Department of Pathology, Geisel School of Medicine at Dartmouth, Hanover NH 03756, USA; 3 Department of Surgery, Geisel School of Medicine at Dartmouth, Hanover NH 03756, USA; 4 Beckman Laser Institute, University of California Irvine, Irvine CA 92617, USA; 5 Center for Systems Biology at Harvard Medical School/MGH, 185 Cambridge Street, Suite 5.210, Boston MA, USA

**Keywords:** BCS/BCT, Breast-conserving surgery/therapy, Near-infrared spectroscopy, Spatial frequency domain imaging, Diagnostic pathology

## Abstract

**Introduction:**

Nationally, 25% to 50% of patients undergoing lumpectomy for local management of breast cancer require a secondary excision because of the persistence of residual tumor. Intraoperative assessment of specimen margins by frozen-section analysis is not widely adopted in breast-conserving surgery. Here, a new approach to wide-field optical imaging of breast pathology *in situ* was tested to determine whether the system could accurately discriminate cancer from benign tissues before routine pathological processing.

**Methods:**

Spatial frequency domain imaging (SFDI) was used to quantify near-infrared (NIR) optical parameters at the surface of 47 lumpectomy tissue specimens. Spatial frequency and wavelength-dependent reflectance spectra were parameterized with matched simulations of light transport. Spectral images were co-registered to histopathology in adjacent, stained sections of the tissue, cut in the geometry imaged *in situ*. A supervised classifier and feature-selection algorithm were implemented to automate discrimination of breast pathologies and to rank the contribution of each parameter to a diagnosis.

**Results:**

Spectral parameters distinguished all pathology subtypes with 82% accuracy and benign (fibrocystic disease, fibroadenoma) from malignant (DCIS, invasive cancer, and partially treated invasive cancer after neoadjuvant chemotherapy) pathologies with 88% accuracy, high specificity (93%), and reasonable sensitivity (79%). Although spectral absorption and scattering features were essential components of the discriminant classifier, scattering exhibited lower variance and contributed most to tissue-type separation. The scattering slope was sensitive to stromal and epithelial distributions measured with quantitative immunohistochemistry.

**Conclusions:**

SFDI is a new quantitative imaging technique that renders a specific tissue-type diagnosis. Its combination of planar sampling and frequency-dependent depth sensing is clinically pragmatic and appropriate for breast surgical-margin assessment. This study is the first to apply SFDI to pathology discrimination in surgical breast tissues. It represents an important step toward imaging surgical specimens immediately *ex vivo* to reduce the high rate of secondary excisions associated with breast lumpectomy procedures.

## Introduction

Optimal local management of breast cancer has been hindered by an inability to assess tumor-margin status intraoperatively, predominantly because frozen sections are limited by freezing artifacts in adipose tissue [1,2], and sensitivities reported for touch-preparation cytology have been inconsistent [3]. Breast-conserving therapy (BCT), which includes local tumor excision followed by moderate-dose radiation therapy, is the standard of care for patients with early invasive breast cancer (stage I and II) and for patients with advanced disease whose tumor burden has been successfully reduced with neoadjuvant chemotherapy [4,5]. It was the treatment of choice for nearly 75% of the approximately 300,000 new breast cancer patients diagnosed in 2011 [6]. Prospective, randomized trials have demonstrated that BCT survival rates are equivalent to those of mastectomy when surgical margins are negative for residual disease [4,5], but positive margins have been associated with an increased risk of local recurrence [7-9] and mortality [10]. Consequently, reexcision of a margin is the standard of care if invasive tumor is detected at the surface of the resected specimen, and also occurs at Dartmouth-Hitchcock Medical Center (DHMC) when ductal carcinoma *in situ* (DCIS) is found within 2 mm of the inked tissue surface. Wider margins are examined for DCIS because it can be multifocal and interspersed within normal tissues [11]. At DHMC in 2010, invasive cancer was detected at the margin, and *in situ* cancer was discovered at or within 2 mm of the margin in 20% and 23% (respectively) of patients treated with BCT for invasive carcinomas (*n* = 129), yielding a 43% reexcision rate (36% actually reexcised). If residual tumor could be detected at one or more margins during the initial surgery, the surgeon could take directed, additional tissue before closing.

Neoplastic processes, from early dysplasia to advanced-stage infiltrating tumors, perturb tissue ultrastructure and thereby alter its optical-scattering spectrum [12,13]. The scattering spectrum can distinguish tissue pathologies when the optical signal is sampled locally [14-18] or filtered by using polarization techniques [19-21] to minimize the collection of multiply scattered light. Localized methods, such as optical coherence tomography (OCT) [22], Raman spectroscopy [23], and confocal sampling [24,25], have been applied to surgical-margin assessment, but they are fundamentally limited in depth sampling by scattering attenuation in tissue. In most realizations, the microscopic field of view (FOV) is too small to evaluate surgical specimens wholly, so they have the same margin-sampling limitations as does conventional histopathology. Resected tissues may include lesions up to 5 cm in diameter, surrounded by a targeted layer of grossly normal breast that can be as thick as 1 cm. Wide-field imaging with localized sampling has recently been realized through multiplexed arrays of probes [17,18,26,27]; these approaches still rely on discrete sampling to form images and thereby incompletely assess disease extent, multifocality, and tumor heterogeneity. Raster-scanning techniques [16-18,28] support high-resolution sampling to assess tumor heterogeneity, but have limited field-of-view and speed to allow scanning the complete surgical specimen in surgical settings. Ideally, the complete surgical specimen would be evaluated (in a noncontact manner) without sacrificing sensitivity to tumor-specific features in the scattering spectrum. Planar spectral imaging techniques have only recently been tailored to surgical resection guidance [29-31] and sentinel lymph node (SLN) mapping [30,32,33], largely because of the explosive development of molecularly specific NIR probes [34]. However, most methods rely on diffuse light transport, which can be insufficient to resolve important morphologic transformations that have dimensions comparable to the optical wavelength [35], because its spatial resolution is limited by light scattering in tissues [36].

SFDI, a planar-imaging modality pioneered by investigators at the University of California at Irvine and commercialized by Modulated Imaging Inc. for biologic imaging at spatial resolutions between coherent and diffuse optical-imaging techniques, was applied here for wide-field, tissue-type discrimination in nearly 50 surgical breast lesions. SFDI quantitatively resolves subsurface tissue absorption and scattering by analyzing the spatial-modulation transfer function (s-MTF) at multiple NIR wavelengths [37]. Planar, structured light patterns improve signal localization and enable selective depth sampling [38]. Recovered optical parameters are surface-weighted, which may have added value for surgical-margin assessment, where the goal is to detect malignant transformations in the outer millimeters of resected tissue. In this contribution, supervised learning and feature-selection algorithms were implemented to automate spectral discrimination of pathologies in intact, surgical tissues examined with SFDI and to optimize future development of spectroscopic tools for margin assessment.

## Methods

### Spatial frequency domain imaging (SFDI)

A compact SFDI system (purchased from Modulated Imaging Inc., Irvine, CA) illuminated breast surgical-resection specimens with a harmonically modulated, planar source at four NIR wavelengths (658, 730, 850, and 970 nm) [37]. Structured light patterns were projected onto each tissue surface at 30 spatial frequencies uniformly distributed between 0 mm^-1^ and 0.33 mm^-1^ by using high-power light-emitting diodes (LEDs), a projection system, and a digital micro-mirror device. The projector and camera subsystems were described in previous publications [37,39] and were fully integrated in a portable platform mounted on a z-axis post. In total, 360 images were acquired per tissue (30 spatial frequencies × 3 phase offsets × 4 wavelengths) in approximately 10 minutes. Data were simultaneously acquired over the full field in a noncontact geometry, in which the acquisition field of view (FOV) was determined by magnification of the illumination and collection optics, here optimized to image a 5.5-inch × 7.5-inch area. A 12-bit CCD-based camera, co-registered with the projector, collected diffusely reflected light at a 696 × 520 pixel resolution with 2 × 2 pixel binning (full pixel resolution of the camera is 1,392 × 1,040). Fundamentally, however, the detected spatial resolution is limited by the physics of light transport in tissue, and contrast-detail analysis was previously performed to evaluate the minimized size of detectable scattering contrast in tissue-simulating phantoms [40]. A three-phase demodulation scheme was then used to extract the frequency-dependent modulation amplitude and to remove noise and ambient light from the imaged field [41]. The spatial frequency of the illumination pattern integrates optical parameterization with effective sampling depth, in which higher spatial frequencies attenuate more rapidly and thereby sample superficial tissue volumes. The effective sampling depth is also determined by the tissue optical properties. Theoretically, probing depths of 1 to 8 mm are expected in tissues; experimentally, we demonstrated detection of scattering inclusions up to depths of 1.5 mm in tissue-simulating phantoms by using the higher spatial frequencies used in this study [40]. At each imaging session, the modulation amplitude of a Siloxane titanium dioxide (TiO_2_) reflectance standard with known optical properties was imaged to calibrate spatial nonuniformity in the illumination and imaging systems. A 1-mm uniform height offset was erroneously, but systematically, introduced between measures of the tissue and reference standard. The offset did not affect tissue discrimination and resulted in negligible scaling of the modulation amplitude because reflectance decays with distance, *h*, according to an inverse square law (the distance between the CCD lens and tissue was ~120 mm $h_{2}^{2}/h_{1}^{2}=119mm^{2}/120mm^{2}≅1$[42].

Optical parameters were estimated by minimizing the residual sum of squares between the measured modulation amplitude and its forward simulation, according to a scaled Monte Carlo model of light transport [37]. Monte Carlo solutions were stored in a look-up table (LUT) to reduce the computational burden associated with iterative estimation of spectral parameters [39]. Subsequently, spectral parameters were fit according to absorption by the endogenous tissue chromophores oxygenated-hemoglobin, deoxygenated-hemoglobin, and water, and the wavelength-dependence of light scattering, and produced estimates of scattering amplitude (*A*), scattering slope (*b*), total hemoglobin concentration (*HbT*), percentage hemoglobin oxygenation (*%O*_*2*_), and percentage water (*%H*_*2*_*O*). Data-acquisition and analysis methods are discussed more comprehensively in a companion article [39].

### Imaging protocol for the surgical specimens

The ability of SFDI to distinguish histopathology, here used as the diagnostic gold standard, was evaluated at the cut surfaces of 47 surgical breast tissues. In this HIPAA-compliant, prospective study, approved by the Institutional Review Board for the protection of human subjects at Dartmouth, written informed consent was not required for participants, although an Information Sheet detailing the study was provided with an opt-out provision. All of the study patients were female. A breakdown of the pathologic characteristics of the imaged cancers is provided in Table 1. In addition to these 27 biopsy-proven cancers, 11 fibroadenomas and nine normal (including fibrocystic disease) breast cases were imaged. At Dartmouth, the breast-resection specimens are inked with six pre-assigned colors to preserve orientation within the breast, so that, if it is found by pathologic evaluation that the margins are involved by tumor, the patient is called back to undergo a second resection of those specific margins rather than the entire surgical cavity.

**Table 1:** Summary of clinical and pathology data for study participants with a cancer diagnosis

**Patient number**	**Age (years)**	**Tumor size (cm)**	**Tumor type**	**Tumor grade**	**ER (IHC score)**	**PR (IHC score)**	**HER2neu ratio (FISH)**	**Specimen size (cm)**
1	51-55	0.3	DCIS	Low	Pos	Pos	N/A	12.8 × 9.5 × 1.7
2	56-60	7.0	IDCa	High	Neg	Neg	1.1	10.5 × 8.5 × 3.0
3	51-55	2.2	IDCa	Low	Pos	Pos	1.2	7.0 × 6.0 × 1.7
4	61-65	2.2	IDCa	Int	Pos	Pos	1.3	7.0 × 6.8 × 1.3
5	56-60	1.8	IDCa	Int	Pos	Pos	1.0	6.0 × 5.6 × 2.0
6	56-60	2.8	IDCa	High	Neg	Pos	1.1	7.0 × 5.0 × 1.6
7	56-60	1.8	IDCa	Int	Pos	Pos	1.1	9.0 × 7.5 × 2.2
8	56-60	10.0	IDCa**	High	Neg	Neg	6.0	23 × 15 × 6.0
9	56-60	2.0	IDCa**	Int	Neg	Neg	1.0	15 × 15 × 4.0
10	66-70	1.6	IDCa	High	Pos	Neg	1.2	8.5 × 7.0 × 2.0
11	76-80	5.0	DCIS	High	Pos	Pos	N/A	30 × 20 × 5.0
12	56-60	1.6	IDCa	Int	Pos	Pos	1.1	24 × 13 × 3.0
13	56-60	0.8	IDCa**	Int	Pos	Neg	9.0	13.5 × 9.0 × 2.0
14	56-60	6.5	IDCa**	High	Pos	Pos	1.0	22 × 15 × 4.5
15	61-65	5.0	ILCa**	Int	Pos	Pos	1.1	22 × 20 × 5.0
16	71-75	2.5	IDCa	High	Pos	Pos	1.1	7.3 × 7.0 × 3.0
17	61-65	0.4 1.5	IDCa DCIS	Int	Pos	Pos	1.0	9.0 × 8.5 × 2.3
18	56-60	0.5	IDCa	Int	Pos	Pos	1.1	5.0 × 5.0 × 1.3
19	61-65	1.6	IDCa	High	Pos	Pos	1.2	22.5 × 19 × 3.5
20	51-55	2.1	IDCa	Low	Pos	Pos	1.0	7.0 × 5.6 × 1.7
21	61-65	2.4	IDCa	Int	Pos	Pos	1.2	6.7 × 5.6 × 2.0
22	36-40	3.0	IDCa	Int	Pos	Pos	1.2	23 × 23 × 4.0
23	41-45	12.0	IDCa	Int	Pos	Pos	1.1	21 × 18 × 5.0
24	71-75	1.5	IDCa	High	Pos	Pos	1.2	6.0 × 5.0 × 1.6
25	51-55	2.5	IDCa	High	Pos	Pos	1.0	7.0 × 6.5 × 1.4
26	61-65	3.5	IDCa	High	Pos	Neg	1.1	7.5 × 5.0 × 2.7
27	46-50	8.0	IDCa	Int	Pos	Pos	1.2	30 × 29 × 6.0

Tumor size before Rx, maximum dimension for tumor size from pretreatment MRI scan; tumor type: IDCa, infiltrating ductal carcinoma; IDCa**, after chemoRx; ILCa, infiltrating lobular carcinoma. Tumor grade: low, low grade; int, intermediate grade; high, high grade. ER, estrogen-receptor protein; PR, progesterone-receptor protein; IHC score, immunohistochemical semiquantitative score: Pos, (positive) ≥15% IHC staining; Neg (negative), 0 IHC staining. HER2neu ratio, HER2neu gene analysis by fluorescence *in situ* hybridization (FISH) compared with a normal control, ratio < 2.0 is normal expression, ratio 2 to 4 is equivocal expression, and ratio >4.0 is gene overexpression.

The fresh, inked specimens, from patients who did not decline this research use of their tissues, were transported immediately to the Department of Pathology. By standard protocol, all of the specimens were “bread loafed” in their entirety into consecutive slices up to 0.5 cm in thickness, and, for this study, one face of one slice representing the lesion and its relation to the nearest margin was imaged. No surgical margin was imaged *en face*, given the required orientation inking by the surgeon. The exact area of tissue imaged in one slice was outlined with four inked pins pushed through the tissue block. The tissue was returned to Pathology after imaging, within 30 minutes of resection, for standard histologic processing. The imaged slices were formalin-fixed with the pins *in situ* before being removed for usual processing. The tiny inked pinholes survive processing and are evident in the histologic sections of that imaged tissue, facilitating optimal registration with the histologic findings. The effects of tissue shrinkage after processing and sectioning distortion were minimized by ensuring optimal formalin concentration and fixation time in accordance with College of American Pathologist (CAP) best practices. Specimen imaging did not affect procedure time in the operating room, processing time in the Department of Pathology, or the content and time to verification of the final pathology report.

**Figure 1:**
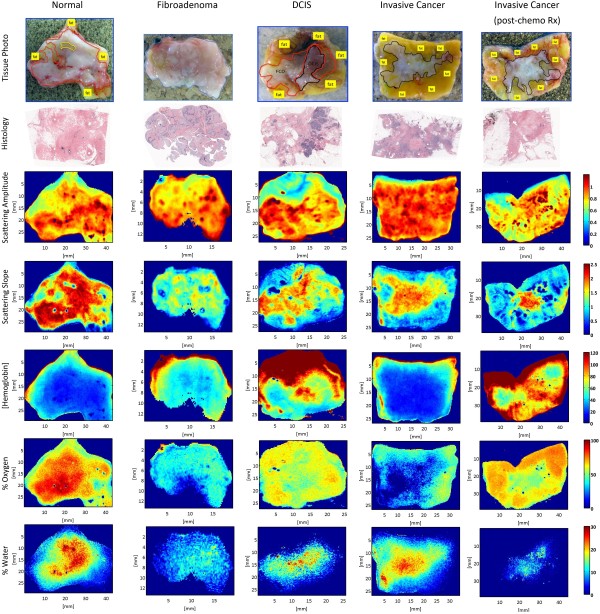
**Representative spectral parameter maps for tissue subtypes.** Spectral parameter maps corresponding to the pathology subtypes: normal (including fibrocystic disease) (red outline), fibroadenoma (blue outline), DCIS, invasive cancer and partially treated invasive cancer after neoadjuvant chemotherapy (all black outline), and fat (yellow outline or label). Row 1 is a tissue photograph of the cut face of one slice of the specimen with the lesion; row 2 is the corresponding histology; row 3 is the scattering-amplitude maps; row 4 is the scattering slope maps; row 5 is the hemoglobin concentration maps; row 6 is the percentage oxygenated hemoglobin maps; and row 7 is the percentage water maps.

The findings in histologic sections representing the imaged areas were included in the final pathology report. Figure 1 illustrates co-registration between photographs of the cut face of one slice in the specimen, with the lesions outlined in diagnosis-defined colors (row 1), corresponding histopathology (row 2), and spectral images (rows 3 to 7). An experienced breast pathologist (WAW) outlined tissue areas containing target breast lesions on digital photographs of each specimen, guided by co-registered hematoxylin and eosin (H&E)-stained tissue sections from the imaged surface. Affine transformations were manually performed in Photoshop to convert the outlined tissue areas into masks for region-based image analysis in MATLAB (The MathWorks Inc., Natick, MA, USA).

In total, 48 specimens from 47 patients were consecutively imaged in this study; one was excluded from analysis because surgical inks bled into the primary tissue field. Surgical inks, sometimes observed at the tissue edge, were avoided during region of interest (ROI) selection and masked according to the R^2^ coefficient of determination (pixels excluded if *R*^*2*^ < 0.95). Spatial frequency- and wavelength-dependent reflectance measurements were evaluated on a pixel-by-pixel basis in 59 regions of interest (>265,000 spectral pixels). Discrimination was assessed between benign and malignant pathologies and between the benign pathology subtypes (normal and fibroadenoma), and the malignant pathology subtypes (DCIS, invasive cancer, and partially treated invasive cancer after neoadjuvant chemotherapy), as listed in Table 2. In this analysis, fibrocystic disease was grouped with normal breast pathologies. Tumors treated with neoadjuvant chemotherapy before surgical resection (*n* = 5) were imaged to observe how chemotherapy alters tissue optical properties, the better to inform diffuse tomographic monitoring of treatment response to therapy [43].

**Table 2:** Summary of tissue subtypes imaged

**Spatial frequency domain imaging (47 patients)**			
**Diagnosis**	**ROIs**	**R**_**d**_**(f**_**x**_**, λ)**	
Normal/Fibrocystic (NOR)^a^	22	109,841	Benign 170,158
Fibroadenoma (FA)^a^	11	60,317	
Ductal carcinoma *in situ* (DCIS)	4	8,487	Malignant 94,916
Invasive cancer (INV)	17	63,552	
Invasive cancer, treated (INV, Rx)	5	22,877	
Totals	59	265,074	

Total numbers of ROI and modulated reflectance spectra, *R*_*d*_(*f*_*x*_, *λ*), assessed per diagnostic class. Benign pathologies analyzed include normal or fibrocystic tissues and fibroadenomas (^a^). Malignant pathologies analyzed include ductal carcinoma *in situ*, invasive cancer, and treated invasive cancers.

### Histopathology and immunohistochemical correlates

Spectral maps were associated with morphologic and immunohistochemical markers identified in adjacent, stained sections of the tissue, cut in the exact geometry imaged *in situ*. Possible correlates between immunohistochemical measures and spectral parameters were evaluated according to the Pearson correlation coefficient. CD31 (platelet endothelial cell adhesion molecule) was used to stain immunohistochemically the preexisting endothelial cells as a general indicator of normal vasculature, even though malignant vessels have been reported to retain this antigen [44]. CD105 (Endoglin), thought to antagonize the inhibitory effects of transforming growth factor-beta (TGF-β) on cell proliferation and capillary formation [44], was used to stain immunohistochemically a transmembrane glycoprotein expressed predominantly on the surface of highly proliferating endothelial cells, as an indicator of tumor angiogenesis. Cytokeratin CK5D3 was used as a marker for breast epithelium (benign and malignant). All tissue was fixed in 10% buffered formalin (Biochemical Science Inc, Swedesboro, NJ, USA), dehydrated through graded alcohols, and paraffin embedded. Tissue sections (4 μm) were coated with adhesive (Sta-on; Surgipath Medical Industries, Richmond, IL, USA), mounted on glass slides, and stained with H&E for initial review. Tissue sections were air-dried for at least 30 minutes and then loaded onto a Leica Bond Max automated immunostainer. Here, the sections were baked (30 minutes at 60°C), dewaxed for 30 minutes at 72°C, rinsed with alcohol, and washed in Bond wash buffer. Antigen retrieval was accomplished with Bond epitope Retrieval 2, Ar9640 (pH 8.9 to 9.1) for 20 minutes at 100°C; followed by cooling for 12 minutes and a rinse in Bond wash buffer. All dewaxed, antigen-retrieval, and detection reagents were supplied by Leica Microsystems (Bannockburn, IL, USA). The primary antibodies were incubated for 15 minutes: CK5D3 at 1:100 (Biogenix, Fremont, CA, USA), CD31 at 1:50 (Dako, Carpinteria, CA, USA), and CD105 at 1:60 (Vector, Burlingame, CA, USA). Diaminobenzidine was applied for visualization with a hematoxylin counterstain.

### Quantitation of immunomarkers

Using Media Cybernetics™ Image Pro (Media Cybernetics, Bethesda, MD, USA) and automated stage control bundled software (Surveyor© Automated Specimen Scanning stage control bundled software (Objective Imaging Ltd., Cambridge, UK), whole H&E-stained slides and CD105 and CD31-immunostained slides, representing the imaged areas of lesional breast tissue, were digitally scanned at high resolution and montaged. Mean vessel density (MVD) was quantified according to the areas of CD31-positive (preexisting vasculature) or CD105-positive (tumor-induced) vessels, and segmented in pseudo-color, as a percentage of the total slide area. Mean vessel area (MVA) was determined from the combined areas of CD31-positive or CD105-positive blood vessels, segmented in pseudo-color, and measured in square micrometers. The epithelial component of each imaged area was defined as the percentage of the total slide area showing positive immunostaining for CK5D3 and segmented in pseudo-color. On the same slide, the morphologically distinctive white spaces representing lipocytes (emptied of lipid as a result of fixation and tissue processing) were also segmented in pseudo-color as a percentage of the total slide. The remaining percentage of the slide represented connective tissue stroma. The diagnostic ROIs were selected by WAW, a board-certified surgical pathologist with 15 years of expertise in breast pathology and image analysis. The image thresholding and processing were performed by MCS, a technologist with 6 years of expertise in the Image Pro software.

### Automated discrimination of breast pathologies

A nearest-neighbor learning algorithm was implemented to automate spectral discrimination of benign and malignant pathologies, and all pathology subtypes listed in Table 1; performance was evaluated by using a threefold cross-validation [45]. All data were randomly divided into three nonoverlapping sets, with an equal number of reflectance spectra per diagnostic class per set. Two of these sets were used for training, and the third set was used for validation. Training pixels were associated with a known diagnosis, according to the pathologist’s demarcation of lesions; the diagnosis of each validation point was also identified by the pathologist, but remained blind to the classifier to evaluate its performance. Classification error was taken to be the percentage of misclassified pixels in the validation set, where a misclassification means that the diagnosis assigned by the classifier did not match the diagnosis provided by the pathologist. Performance metrics were quantified 3 times for all possible permutations of the training and validation sets, and reported values were the average of these three executions. The nearest-neighbor algorithm assigned each unclassified parameter set to the majority diagnosis of its *k* nearest neighbors found in the training space by an efficient k-dimensional tree-search algorithm [46]. A whitening transformation was applied to all spectral parameters before diagnostic discrimination to prevent amplitude weighting. Additionally, outliers were removed from the training set according to their interquartile fractions (comprising 5% of the total data set). Query points were never marked as outliers, because this information was not known *a priori*.

Receiver operating characteristic (ROC) analysis was used to optimize classifier sensitivity and bias as a function of nearest-neighbor number, *k*. Confidence intervals (α = 0.05) for the sensitivity and specificity were computed according to the Yates χ^2^ interval [47].

### Optical parameter ranking

An iterative search algorithm, sequential floating forward selection (SFFS), was implemented to rank the contribution of each spectral parameter to tissue-type discrimination. The Bhattacharyya statistical distance, *J*_*ij*_, was used to measure the separation between two diagnostic classes (i,j) [48,49] and was generalized to all spectral parameters (n = 5) by:

$$J=\sum_{i=1}^{5}\sum_{j=1}^{5}P_{i}P_{j}J_{\mathrm{ij}}$$

Here, P_i_ represents the prior probability of class i determined by its fraction of pixels in the training set. The search algorithm ranked all parameters in order of the contribution to diagnostic-class separability.

## Results

### Optical biomarkers of breast pathologies

**Figure 2:**
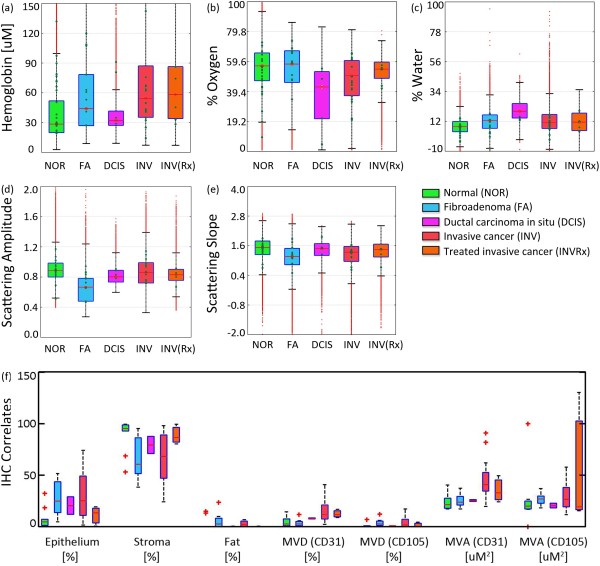
**Diagnostic distributions of spectral parameters and immunohistochemistry correlates. (a**-**e)** Boxplots of recovered spectral parameters for all tissues per pathology subtype, indicated by color: normal (including fibrocystic disease) (green), fibroadenoma (blue), ductal carcinoma *in situ* (pink)*,* invasive cancer (red), and partially treated invasive cancer after neoadjuvant chemotherapy (orange)*.***(f)** Corresponding boxplots of immunohistochemical measures of percentage of stroma, epithelium, and fat, and CD31-positive, CD105-positive mean vascular density (MVD) and area (MVA). Box plot red bars indicate the population median; green dots indicate the mean value per patient ROI; and red crosses indicate outliers.

Examples of optical maps co-registered with digital photographs of the surgically resected tissues are shown in Figure 1 for each lesion type evaluated. Boxplots in Figure 2 quantify the extensive distribution of optical and immunohistochemistry markers for all reflectance measures evaluated on a pixel-by-pixel basis (>265,000 measurements); mean values per patient are plotted as green dots that overlie the boxplots. Median values were compared to identify optical signatures specific to each diagnostic class, and interquartile fractions reflect the inherent heterogeneity observed within and between tissues. Median values were significantly different within 95% confidence limits for nonoverlapping notches. Distributions of the scattering amplitude and scattering slope showed less variance than functional absorption parameters, like hemoglobin concentration and oxygenation status, because the morphologic transformations that determine light scattering occur on submicron and even subnanometer length scales [50,51]. Absorption parameters typically signal broader functions, and their distributions were characterized by greater variance because of surgery-induced artifacts like vascular disruption, eradication of the tissue’s oxygen supply, and eventual tissue dehydration. Efforts were made to image all tissues within the same time window and to avoid sampling of cauterization and hemorrhage, but limited artifacts are plausible. Surgical inks at the edge of the resected specimens may have also contributed to outliers with artificially high hemoglobin concentrations. This variability was also detected immunohistochemically in the subsequently fixed tissues, as shown in Figure 2f. However, SFDI assess the full tissue surface, accounting for inherent biologic heterogeneity, so that on average, functional biomarkers may strengthen tissue-type discrimination.

The distributions in Figure 2 show that observed hallmarks of normal or fibrocystic pathologies included a heightened scattering amplitude and slope, as compared with malignant pathologies. Lower scattering amplitude was observed in fibroadenomas, which contain clearly encapsulated and abundant stromal cellularity, confirming diagnostic trends established in previous studies of localized spectroscopic scattering from breast tissues [16]. Higher hemoglobin and reduced oxygenation values were observed in invasive cancers, as compared with the benign pathologies. DCIS was spectrally distinguished from other pathologies by decreased oxygenation and increased water levels, suggesting heightened metabolism and a proportional propensity for more surrounding fibrous stroma. However, a small DCIS patient population (*n* = 4) and spectral parameterization limited interpretation of this signature (fits were based on the extinction of pure, unbound water and did not include absorption by adipose tissue). Neoadjuvant chemotherapy increased the scattering slope when comparing its untreated, invasive counterparts, regardless of response to therapy. An increase in scattering slope suggests cell shrinkage, given its increased propensity for Rayleigh-type scattering.

Increased oxygenation levels were also induced in tumors partially responsive to chemotherapy and could be valuable indicators for therapy monitoring. Many aggressive tumors thrive in hypoxic environments [52], so increased oxygenation levels suggest a return to the normal phenotype.

Pairwise correlations between spectral parameters and quantitative immunohistochemistry are summarized in Table 3. Collagen in the extracellular stroma demonstrated a strong, positive correlation with scattering slope because the small fibrils that form collagen fibers further enhance Rayleigh-type scattering [53]. In contrast, a strong negative correlation was observed between tissue epithelial content and the scattering slope. Malignant tissues expressed greater levels of angiogenic vessels and preexisting vascular endothelium. This net growth in vascularity was detected spectroscopically by increased total hemoglobin, but no optical distinction was made between CD105-specific and CD31-specific vasculature. Immunohistochemical measures were sampled in discrete locations about the tissue surface, limiting its complete representation of ROIs.

**Table 3:** Spectral-immunohistochemical correlates

	**Scattering amplitude**	**Scattering slope**	**Hemoglobin concentration**	**% Hemoglobin oxygenation**	**% Water**
% Epithelium	−0.06	***−0.37***	***0.27***	−0.11	−0.21
% Stroma	0.06	***0.33***	***−0.28***	0.09	***0.25***
% Fat	−0.02	−0.01	0.21	0.02	***−0.28***
Total mean vessel density	0.18	***−0.42***	***0.37***	−0.19	0.00
Total mean vessel area	0.01	***−0.34***	0.12	***−0.28***	0.02

The Pearson correlation coefficient is tabulated for spectral-immunohistochemical pairs. Negative and positive correlations >20% are highlighted in italics.

### Pathology discrimination in resected breast tissues

**Figure 3:**
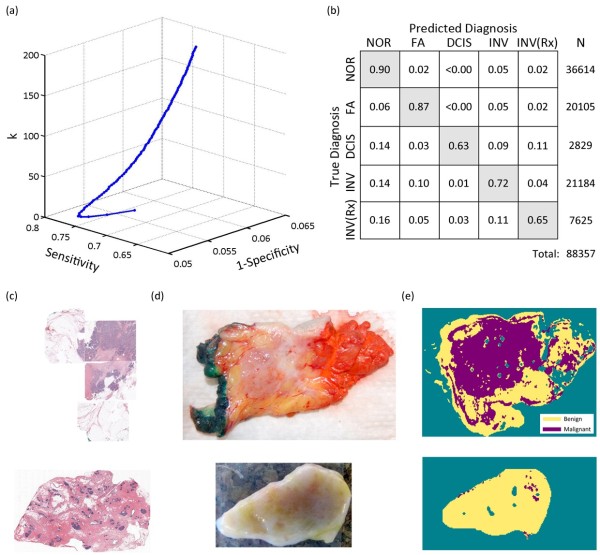
**Optimization and performance of the nearest-neighbor classifier for diagnostic discrimination. (a)** Nearest-neighbor number optimization by using the receiver operating characteristic curve for discrimination between all pathology subtypes. **(b)** A confusion matrix showing the true and predicted diagnosis for all spectroscopic measures according to the nearest-neighbor classifier, presented as a percentage of the total number of diagnosed pixels (N). A multiparametric diagnostic map for a malignant (row 1) and benign (row 2) tissue is illustrated **(c** through **e)**; the patient histology is shown in column **(c)**, a photograph of the tissue imaged by SFDI is shown in column **(d)**, and the patient-specific diagnostic map generated by the classifier is shown in column **(e)**.

Spatial frequency- and wavelength-dependent reflectance measures were applied to the discriminant classifier on a pixel-by-pixel basis, and separation of all tissue subtypes was achieved with 82% accuracy. Figure 3a shows that diagnostic sensitivity increased with nearest neighbor number at the cost of specificity or bias. ROC curves revealed that 11 and nine nearest neighbors optimized separation of benign and malignant pathologies and all pathology subtypes listed in Table 1, respectively. The confusion matrix in Figure 3b explicitly reflects the true and predicted diagnosis as a percentage of total number of diagnosed pixels. True positives per tissue type are along the diagonal, and misclassifications are off-axis; showing that, for example, 16% of treated invasive tumors were misclassified as normal. Figure 3c through e shows a false-color map of the multiparametric diagnosis co-registered with pathology for a malignant and benign tissue to illustrate application of the classifier to a patient diagnosis. Diagnostic performance is summarized in Table 4, which reports the sensitivity, specificity, positive predictive value (PPV), negative predictive value (NPV), and accuracy observed per tissue type. Discrimination of benign from malignant pathologies was highly specific (93%) and reasonably sensitive (79%), although sensitivity was limited in underrepresented pathologies like DCIS and residual invasive cancers after neoadjuvant chemotherapy (61% to 66%). Discrimination between benign and malignant pathologies was more accurate (88%) because these distribution sizes were more equally represented in the classifier training set. Ultimately, the surgeon can moderately regulate diagnostic sensitivity and specificity by varying the number of nearest neighbors used by the classifier to generate a diagnosis, as shown in Figure 3a. The NPV (89%) between benign and malignant pathologies was higher than the PPV (86%). In practical terms, surgeons are more interested in a high NPV when high confidence exists that no residual cancer has been left behind. A lower PPV, correlating with a false-positive call on any margin, would mean that the surgeon would take a little more tissue at the appropriate margin at the time of the first surgery. Even if that margin does not subsequently turn out to be positive, this can be justified clinically by the improved cosmetic outcome, the accuracy of immediate margin reexcision, the reduced costs of a single surgical procedure, and patient well-being by avoiding a second, separate reexcision procedure. High NPVs were also reported for underrepresented tissue types, but these values appear inflated when the number of true negatives significantly outweighed the number of true positives.

**Table 4:** Summary of diagnostic performance

	**NOR**	**FA**	**DCIS**	**INV**	**INV (Rx)**	**Benign versus malignant**
Sensitivity	0.90	0.87	0.61-0.65	0.71-0.73	0.64-0.66	0.79
Specificity	0.89	0.95	0.99	0.94	0.97	0.93
PPV	0.85	0.84	0.76	0.80	0.70	0.86
NPV	0.93	0.96	0.99	0.91	0.97	0.89
Accuracy	0.82					0.88

Summary of diagnostic performance, sensitivity, specificity, positive predictive value (PPV), negative predictive value (NPV), per tissue-type for discrimination between all tissue subtypes, normal (NOR), fibroadenoma (FA), ductal carcinoma *in situ (DCIS),* invasive cancer (INV), and treated invasive cancer (INV(Rx)), and for discrimination between benign and malignant pathologies; the 95% confidence interval is reported for sensitivity and specificity values when the confidence interval range is >1%.

### Diagnostic value of optical parameters

The SFFS algorithm ranked the region-averaged (1) scattering amplitude, (2) percentage water, (3) total hemoglobin concentration, (4) the scattering slope, and (5) percentage oxygen, as most significant to tissue discrimination in that order. The scattering amplitude was most valuable to pathology discrimination, even through qualitatively, the scattering slope appeared to localize better with suggestive fibroglandular lesions. The scattering slope was potentially undervalued by the limitations of pathology co-registration. Percentage oxygenated hemoglobin was least valuable to a diagnosis, mainly because of *ex vivo* fluctuations in hemoglobin oxygenation status. Bydlon [54] showed that percentage of oxygenated hemoglobin varies nonlinearly within 30 minutes of resection, but other spectral parameters, like the scattering coefficients and total hemoglobin concentration, remained relatively stable after excision.

## Discussion

To achieve the best survival outcomes for patients with breast cancer, the goal for BCT is to completely resect the tumor with negative margins and simultaneously take a minimum of tissue to preserve the shape of the breast [4,5]. At Dartmouth and elsewhere, inking the specimen with multiple colors to document better its orientation within the breast ensures that if a margin were found to be involved by tumor pathologic examination, a more directed approach to additional tissue removal at a second resection procedure is associated with lower recurrence rates and better cosmesis [55]. This reexcision would be better performed at the time of the first surgery rather than waiting at least 48 hours, as is currently the case. Although a preliminary pathological analysis can be performed while the surgeon waits in the surgical suite, these current methods are far less accurate [1-3] and not amenable to surgical workflow or operating room efficiency. As a result, the initial surgery is concluded, and patients recover before the final pathological margin assessment. Effective and efficient patient care demands an intraoperative technique that minimizes the need for reexcisions and maximizes the cosmetic outcomes by assisting the surgeon in removing the minimum normal tissue around the tumor necessary to ensure a negative margin within a single procedure.

NIR optical parameters imaged by a new planar imaging modality, SFDI, were combined with a discriminant classifier to detect and differentiate breast pathologies *in situ*. Spectroscopic scattering was emphasized because absorption biomarkers varied markedly with the surgical procedure itself, and a feature-ranking algorithm identified the scattering amplitude as most significant to tissue-type discrimination. All images were interpreted according to histopathology for direct clinical relevance. Spectral parameters applied to a discriminant classifier distinguished benign from malignant pathologies with 88% accuracy and the pathology subtypes: normal (includes fibrocystic disease), fibroadenoma, DCIS, invasive cancer, and partially treated invasive cancer after neoadjuvant chemotherapy), were identified with 82% accuracy. Discrimination of benign from malignant pathologies was highly specific (93%) and reasonably sensitive (79%). The precision of spectral-image co-registration with pathology may have also limited the accuracy of discrimination; ROIs were chosen conservatively here because this study was the first to explore SFDI optical-parameter contrast in resected breast tissues. Microscopic co-registration may not be necessary for surgical-margin assessment, in which the ultimate clinical goal is to determine if residual cancer is present at the surface of a resected tissue. The primary advantage of SFDI is near-real-time assessment of intact, surgical specimens, so that decisions about additional margin sampling can be made at the time of primary surgery without damaging the sample. Clinical workflow and the sample remain intact, so that margin status may be confirmed postoperatively by conventional histopathology. Although its modest PPV (0.86 when discriminating between benign and malignant pathologies) could result in a wider primary excision margin, information is gained only at the time of surgery, and a secondary excision would still be avoided. More important, SFDI identifies breast pathologies with a high NPV (0.89 when discriminating between benign and malignant pathologies and 0.91 to 0.99 when discriminating between all pathology subtypes), ensuring that tissues left unresected are truly negative for residual disease. Ultimately, the surgeon has control over the tradeoff between sensitivity and specificity according to the number of nearest neighbors used by the classifier, as shown in the ROC curve in Figure 3. Therefore, decisions about the width of excisional margin could be made in a patient-specific manner.

Application of SFDI to breast surgical margin assessment may be realized soon, given its development as a commercial package and the discriminatory power demonstrated here. Its planar illumination scheme rapidly imaged 48 surgical tissues, and only one was rejected from analysis because ink (applied postoperatively) contaminated the primary imaged field. Image interpretation was highly efficient when using an LUT for parameter optimization and the k-dimensional search tree in the discriminant classifier. Further reductions in data-acquisition time are possible by limiting the number of spatial frequencies sampled; Cuccia *et al.*[37] demonstrated optical parameter recovery in tissue simulating phantoms with just two spatial frequencies. Recent hardware modifications by Modulated Imaging, Inc., enabled acquisition of 15 spatial frequencies at four wavelengths in less than 2 minutes; this has been tested in four fully intact lumpectomy specimens before gross sectioning by pathology [39].

Inking strategies coordinated with the surgeon will be necessary to translate SFDI further to the intraoperative setting. Although inking is necessary for conventional histopathology and to validate new diagnostic techniques, it is not required for intraoperative margin assessment. Ideally, the surgeon would apply the colored inks subsequent to imaging, but before removal of tissue from the imaging platform. The surgeon would be responsible for moving the specimen to and from the imaging platform and additionally could use sutures to safeguard knowledge of its orientation until inks are applied. In that way, feedback on margin status is provided at the time of primary surgery, and the specimen remains intact for conventional histopathology, maintaining the clinical gold standard. In this study, bread-loafed sections of the resected tissue were imaged to enable accurate co-registration with pathology for quantitative assessment of SFDI diagnostic performance, as compared with the clinical gold standard. SFDI images a surface area much larger than the microscopic FOV sampled by a pathologist, so it does not have the sampling limitations encountered in conventional histopathology. An initial demonstration of imaging fully intact surgical margins *en face*, before inking, is reported in our companion systems-analysis article [40]. Edge artifacts caused by surface profile changes were sometimes observed in intact specimens, but a three-phase amplitude demodulation scheme has been implemented to correct for surface profile changes [42]. Future development of SFDI for surgical-margin assessment should focus on enhancing tumor-specific scattering contrast, improving methods for spectral image co-registration with pathology, and automating image processing for near-real-time diagnostic feedback at the time of surgery. Increasing the density of visible-NIR sampling may also improve depth resolution and the quantification of significant absorption signatures like water.

## Conclusions

SFDI combines quantitative spectroscopy with depth sampling appropriate for margin assessment and optimally balances the tradeoffs between wide-field acquisition and signal localization. Most important, it performs diagnostic assessment rapidly and on intact surgical specimens, providing intraoperative feedback to the surgeon. Submillimeter probing volumes limit microscopic resolution, but are here demonstrated to be diagnostically powerful and clinically pragmatic. Planar imaging of NIR optical parameters, in contrast to probing discrete tissue regions by using fiberoptics, comprehensively images known biologic heterogeneity within pathology subtypes. In combination with a discriminant classifier, this wealthy data set readily provides a tissue-type diagnosis. SFDI distinguished benign from malignant pathologies within 47 surgically resected breast tissues with 88% accuracy and discriminated between all pathology subtypes with 82% accuracy. Discrimination of benign from malignant pathologies was highly specific (93%) and reasonably sensitive (79%). Although spectral absorption and scattering features were essential components of the classifier, scattering exhibited lower variance, and the scattering amplitude contributed most to tissue-type separation. The scattering slope was also exquisitely sensitive to stromal and epithelial distributions measured with quantitative immunohistochemistry. Certain clinical challenges, mainly orienting spectroscopic images with pathology and the patient, and integrating data acquisition with immediate processing at the time of surgery, remain to be effected, but here we demonstrated that SFDI can rapidly discriminate between microscopic pathologies at the surface of lumpectomy tissues. This work validates the continued translation of SFDI toward evaluation of surgical specimens immediately *ex vivo* and at the time of primary surgery to reduce significantly the secondary excision rate currently associated with breast lumpectomy procedures.

## Abbreviations

%H2O: Percentage water; %O2: Percentage hemoglobin oxygen; A: Scattering amplitude; b: Scattering slope; BCT/BCS: Breast-conserving therapy/surgery; CAP: College of American Pathologists; CCD: Charge-coupled device; CD105: Endoglin; CD31: Platelet endothelial cell adhesion molecule; DCIS: Ductal carcinoma *in situ*; DHMC: Dartmouth-Hitchcock Medical Center; ER: Estrogen-receptor protein; FA: Fibroadenoma; FISH: Fluorescence *in situ* hybridization; FOV: Field of view; H&E: Hematoxylin and eosin; HbT: Total concentration of hemoglobin; HIPPA: Health Insurance Portability and Accountability Act; IHC: Immunohistochemical; ILCa: Infiltrating lobular carcinoma; Int: Intermediate grade; INV: Invasive cancer; INV(Rx): Treated invasive cancer (residual); K: Nearest neighbor number; LEDs: Light-emitting diodes; LUT: Look-up table; MVA: Mean vessel area; MVD: Mean vessel density; Neg: Negative; NIR: Near-infrared; NOR: Normal; NPV: Negative predictive value; OCT: Optical coherence tomography; Pos: Positive; PPV: Positive predictive value; PR: Progesterone-receptor protein; pre-Rx: Pretreatment; ROC: Receiver operator characteristic; ROI: Region of interest; SFDI: Spatial frequency domain imaging; SFFS: Sequential floating-forward selection; SLN: Sentinel lymph node; s-MTF: Spatial-modulation transfer function; TGF: Transforming growth factor; TiO2: Titanium dioxide; WAW: Wendy A. Wells.

## Competing interests

AML, VK, EJR, MCS, RJB, KDP, BWP, and WAW declare no competing interests. BT and DC report patents, owned by the University of California, that are related to the SFDI technology and analysis methods. The University of California has licensed SFDI technologies to two companies, Caliper/Perkin-Elmer, Inc., and Modulated Imaging, Inc. BT and DC are cofounders of Modulated Imaging. This research was completed without financial support of either company.

## Authors’ contributions

BT and DC provided the original SFDI technology and analysis methods. AML and VK designed, adapted, and analyzed the performance of the new imaging technology. AML evaluated all images and quantified their diagnostic significance. EJR procured the breast tissue and ensured that the clinical standard-of-care for breast-specimen processing and reporting was maintained at all times. RJB talked to the patients about the study, performed the surgery, and ensured that the specimens were transported from the operating room in a timely manner. AML, WAW, EJR, BWP, and KDP participated in the design of the study and drafted the manuscript. MCS performed the quantitative image analysis of the immunohistochemical correlates. All authors read and approved the final manuscript.
